# Venous Thromboembolism Risk Following Total Joint Arthroplasty in Rheumatoid Arthritis Patients Treated with Janus Kinase Inhibitors Versus Tumor Necrosis Factor Inhibitors: A Propensity Score-Matched Cohort Study

**DOI:** 10.3390/jcm15187098

**Published:** 2026-09-13

**Authors:** Rafaay Kamran, Mahad Chaudhary, Mark Herbert, Mathangi Sridharan, Kellyn R. Hori, Daniel Razick, Amy Blackburn, Andrew Jensen, Alexander B. Christ

**Affiliations:** 1 David Geffen School of Medicine, University of California, Los Angeles, 10833 Le Conte Avenue, Los Angeles, CA 90095, USA; 2 Department of Sciences, Stanford University, 450 Jane Stanford Way, Stanford, CA 94305, USA; 3 Department of Orthopaedic Surgery, University of California, Los Angeles, 100 UCLA Medical Plaza, Los Angeles, CA 90095, USA; 4 Department of Orthopaedic Surgery, Nelson Hospital, Tipahi Street, Nelson South, Nelson 7010, New Zealand

**Keywords:** rheumatoid arthritis, total joint arthroplasty, venous thromboembolism, Janus kinase inhibitors, tumor necrosis factor inhibitors

## Abstract

**Background:** Janus kinase (JAK) inhibitors have been linked to higher venous thromboembolism (VTE) rates than tumor necrosis factor (TNF) inhibitors in rheumatoid arthritis (RA) populations, yet it is uncertain whether this risk extends postoperatively after total joint arthroplasty (TJA). **Methods:** A retrospective propensity score-matched cohort study was performed using a large federated network of de-identified electronic health records. Adults with RA undergoing primary total knee or hip arthroplasty were identified, and patients were categorized based on the use of a JAK inhibitor or a TNF inhibitor within 3 months of surgery. Propensity score matching was conducted in a 1:1 ratio based on demographics, comorbidities, and medications. The primary outcome was VTE, defined as deep vein thrombosis or pulmonary embolism occurring from postoperative day 1 through 365 days; patients with VTE prior to this window were excluded. **Results:** Before matching, 305 JAK inhibitor users and 1299 TNF inhibitor users met inclusion criteria; after propensity score matching, 304 patients remained in each cohort, and exclusion of patients with prior VTE yielded 284 JAK inhibitor users and 288 TNF inhibitor users for analysis. VTE occurred in 17 JAK inhibitor users (6.0%) and 11 TNF inhibitor users (3.8%), corresponding to a risk ratio of 1.57 (95% confidence interval [CI], 0.75–3.29; *p* = 0.230). Kaplan–Meier analysis demonstrated a hazard ratio of 1.56 (95% CI, 0.73–3.33), with no statistically significant difference between groups (log-rank *p* = 0.248). **Conclusions:** Among RA patients undergoing primary TJA, JAK inhibitor use was not associated with a statistically significant increase in postoperative VTE compared with TNF inhibitor use, although VTE events occurred numerically more often in the JAK inhibitor cohort. These findings do not exclude a clinically relevant increase in postoperative thrombotic risk with JAK inhibitors, and larger studies are required to clarify the magnitude of risk and to inform thromboprophylaxis strategies.

## 1. Introduction

Rheumatoid arthritis (RA) is a chronic systemic inflammatory disease characterized by progressive joint destruction that often necessitates total joint arthroplasty to treat end-stage joint disease, particularly total knee arthroplasty (TKA) and total hip arthroplasty (THA) [1]. Patients with RA who undergo arthroplasty face a heightened risk of perioperative complications, including venous thromboembolism (VTE), driven by persistent systemic inflammation, reduced mobility, and a substantial burden of medical comorbidities [2,3].

Over the past decade, Janus kinase (JAK) inhibitors such as tofacitinib, baricitinib, and upadacitinib have transformed the treatment landscape for RA by providing convenient, orally administered targeted immunomodulation [1,4]. At the same time, accumulating safety data have raised concern about an increased risk of thromboembolic events with these agents. In the ORAL Surveillance trial, in which VTE was a secondary safety outcome, a higher incidence of pulmonary embolism and VTE with tofacitinib versus TNF inhibitors reached nominal significance in the 10 mg twice-daily arm, a dose not approved for RA in most markets, and was observed in a cardiovascular risk-enriched population [5,6]. By contrast, controlled real-world analyses have not consistently reproduced this signal: a comparative cohort study found no significant difference in VTE risk between tofacitinib and TNF inhibitor users [7], and real-world data reported thromboembolic rates largely consistent with background risk except among patients with baseline cardiovascular or VTE risk factors [8].

The postoperative period following TKA or THA represents a uniquely hypercoagulable state, shaped by surgical trauma, transient immobility, and amplified inflammatory responses [3,9]. Standard perioperative protocols in RA increasingly incorporate biologic and targeted synthetic disease-modifying antirheumatic drugs. Current ACR/AAHKS perioperative guidelines recommend holding JAK inhibitors 3 days prior to THA or TKA, primarily to reduce infection and wound-healing complications, but they do not provide specific guidance or risk estimates of postoperative VTE [10]. Therefore, the objective of this study was to evaluate postoperative VTE risk among patients with RA undergoing primary TKA or THA who were treated with JAK inhibitors compared to those treated with TNF inhibitors, hypothesizing that JAK inhibitor users would demonstrate higher VTE rates consistent with non-surgical data.

## 2. Methods

### 2.1. Study Design and Data Source

This retrospective cohort study used the TriNetX Global Collaborative Network (TriNetX, LLC, Cambridge, MA, USA), a federated database of de-identified electronic health records from participating healthcare organizations. The data used in this study were accessed on 11 March 2026 from the TriNetX Global Collaborative Network, which provided access to electronic medical records, including diagnoses, procedures, medications, laboratory values, and demographic data, from approximately 191 million patients across 171 healthcare organizations.

The network enables real-time querying of aggregated patient data while maintaining institutional data privacy. This retrospective study is exempt from informed consent, as it represents a secondary analysis of existing data without direct patient interaction; thus, institutional review board approval was waived. All data are de-identified in accordance with the de-identification standard defined in §164.514(a) of the HIPAA Privacy Rule, with the process attested to by a qualified expert as defined in Section §164.514(b)(1). Reporting of this study adhered to the Strengthening the Reporting of Observational Studies in Epidemiology (STROBE) guidelines.

### 2.2. Patient Selection

Adult patients with rheumatoid arthritis were identified using ICD-10-CM codes M05 and M06. Primary total knee arthroplasty (TKA) and total hip arthroplasty (THA) procedures were identified using CPT codes 27447 and 27130, respectively. A diagnosis of rheumatoid arthritis was required at least 1 day before the index arthroplasty to ensure that RA preceded surgery. Patients undergoing revision arthroplasty were excluded to focus on primary procedures with more homogeneous risk profiles.

### 2.3. Exposure Definition

Patients were classified into two mutually exclusive exposure cohorts according to their antirheumatic therapy within the 90 days prior to the index procedure. The JAK inhibitor cohort included patients treated with tofacitinib, baricitinib, or upadacitinib without any concurrent TNF inhibitor use during the exposure window. The TNF inhibitor cohort included patients treated with adalimumab, etanercept, infliximab, golimumab, or certolizumab without concurrent JAK inhibitor exposure in the same period. Because exposure was ascertained from prescription and administration records within this window, cohort assignment reflects recent treatment rather than confirmed active pharmacologic exposure at the time of surgery, particularly in light of the guideline-recommended perioperative interruption of JAK inhibitors.

### 2.4. Index Event

The index event was defined as the date of the primary TKA or THA. All follow-up for outcomes was anchored to this index arthroplasty date.

### 2.5. Outcome

The primary outcome was venous thromboembolism, defined as a diagnosis of deep vein thrombosis (ICD-10-CM I82) or pulmonary embolism (ICD-10-CM I26). Outcomes were ascertained from postoperative day 1 through 365 days after the index procedure to capture both early and delayed VTE events, particularly given low event rates within shorter postoperative intervals that precluded reliable reporting due to data suppression thresholds within TriNetX. Patients with a documented history of VTE diagnosis prior to the start of this outcome window were not excluded from the cohort construction or propensity score matching. However, for outcome analyses, TriNetX was configured to exclude patients with VTE prior to the time window, thereby restricting analyses to incident postoperative events.

### 2.6. Propensity Score Matching

Propensity score matching was applied in a 1:1 ratio to balance baseline characteristics between the JAK inhibitor and TNF inhibitor cohorts. Covariates included age, sex, race, hypertension, diabetes mellitus, hyperlipidemia, obesity, smoking status, prior VTE, prior myocardial infarction, prior stroke, glucocorticoid use, methotrexate use, anticoagulant use, and antiplatelet use. Covariate balance after matching was evaluated using standardized mean differences, with values less than 0.10 prespecified as evidence of adequate balance.

### 2.7. Data Analyses

Between-group differences in the primary outcome were summarized using risk differences, risk ratios, and odds ratios with corresponding 95% confidence intervals. Time-to-event analyses were performed using Kaplan–Meier methods, and cohorts were compared using the log-rank test. Cox proportional hazards models were used to estimate hazard ratios and 95% confidence intervals. A two-sided *p*-value < 0.05 was considered statistically significant. All effect estimates were derived from the propensity score-matched cohorts and are therefore adjusted for the matched covariates through matching; no additional model-based covariate adjustment was applied within the matched set.

## 3. Results

### 3.1. Study Population

A total of 1299 TNF inhibitor users and 305 JAK inhibitor users met inclusion criteria prior to matching. After propensity score matching, 304 patients remained in each cohort. For outcome analyses, patients with venous thromboembolism prior to the outcome window were excluded, resulting in final analytic cohorts of 288 TNF inhibitor users and 284 JAK inhibitor users.

### 3.2. Baseline Characteristics

After propensity score matching, baseline characteristics were balanced between cohorts, with all standardized mean differences less than 0.10. Mean age was 60.1 ± 12.1 years in the TNF inhibitor cohort and 60.0 ± 11.4 years in the JAK inhibitor cohort. The majority of patients were women (81.3% vs. 82.2%) and White (65.5% vs. 66.4%). Cardiometabolic comorbidities were similar between groups, including hypertension (32.6% vs. 35.5%), diabetes mellitus (10.9% vs. 12.2%), hyperlipidemia (23.0% vs. 24.7%), and obesity (15.1% vs. 16.4%). Concomitant medication use was also balanced, including glucocorticoids (36.2% vs. 39.8%), methotrexate (17.4% vs. 17.1%), anticoagulants (7.2% vs. 8.6%), and antiplatelet agents (7.6% vs. 9.2%) (Table 1).

### 3.3. Primary Outcome: Venous Thromboembolism

Venous thromboembolism occurred in 17 patients (5.99%) in the JAK inhibitor cohort and 11 patients (3.82%) in the TNF inhibitor cohort, corresponding to an absolute risk difference of 2.17 percentage points. The risk ratio for JAK inhibitor use versus TNF inhibitor use was 1.57 (95% confidence interval 0.75–3.29), and the odds ratio was 1.60 (95% confidence interval 0.74–3.49). There was no statistically significant difference between groups (*p* = 0.230) (Table 2).

### 3.4. Kaplan–Meier Analysis

**Figure 1:**
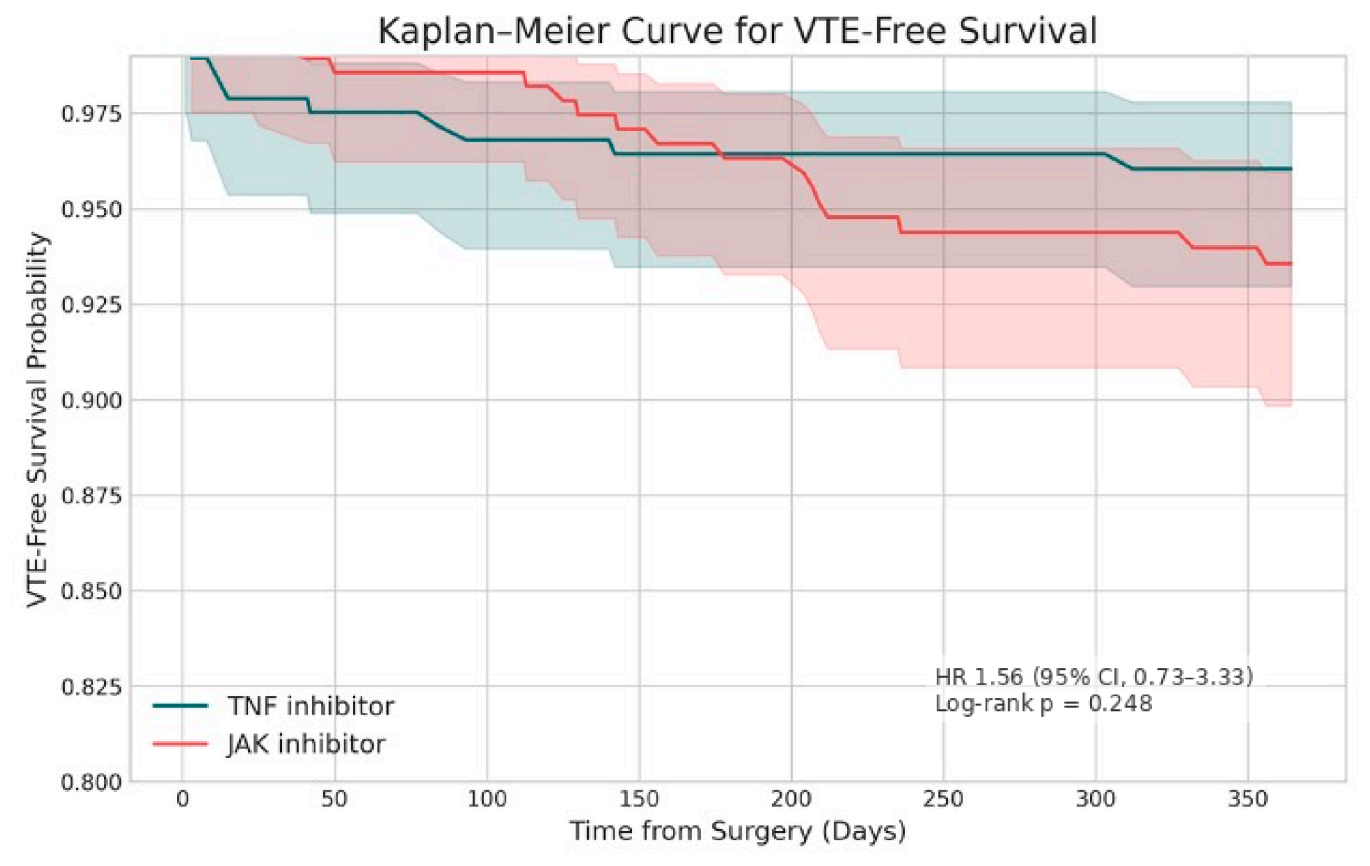
Kaplan–Meier curve for venous thromboembolism-free survival after total joint arthroplasty among patients with rheumatoid arthritis receiving Janus kinase inhibitors versus tumor necrosis factor inhibitors. Shaded regions represent 95% confidence intervals. The hazard ratio is reported for Janus kinase inhibitor use relative to tumor necrosis factor inhibitor use. VTE = venous thromboembolism; JAK = Janus kinase; TNF = tumor necrosis factor; CI = confidence interval.

Kaplan–Meier analysis demonstrated 1-year venous thromboembolism-free survival of 93.6% in the JAK inhibitor cohort and 96.0% in the TNF inhibitor cohort. There was no statistically significant difference between groups (log-rank *p* = 0.248). The hazard ratio for venous thromboembolism comparing JAK inhibitor users with TNF inhibitor users was 1.56 (95% confidence interval 0.73–3.33) (Figure 1).

## 4. Discussion

In this propensity score-matched cohort study, there was no statistically significant difference in postoperative venous thromboembolism risk between patients with rheumatoid arthritis treated with JAK inhibitors and those treated with TNF inhibitors undergoing total joint arthroplasty. Although event rates were numerically higher in the JAK inhibitor cohort, the observed differences were not statistically significant.

These findings are directionally consistent with prior evidence of increased thrombotic risk associated with JAK inhibitors, although controlled real-world analyses have not consistently reproduced this signal [5,6,11]. However, the absence of statistical significance in the present study likely reflects limited statistical power due to low event rates, as well as the wide confidence intervals observed for both risk and hazard estimates. Importantly, the direction of effect was consistent with the prior literature, although the magnitude of difference was small and imprecisely estimated. The absence of a statistically significant difference should not be interpreted as evidence of no increased risk; the wide confidence intervals observed here remain compatible with a clinically meaningful elevation in VTE risk among JAK inhibitor users.

Importantly, both cohorts demonstrated elevated absolute rates of venous thromboembolism compared with the general arthroplasty population, consistent with evidence that RA patients undergoing arthroplasty are at elevated thromboembolic risk [12,13]. This suggests that patients with rheumatoid arthritis undergoing total joint arthroplasty represent a high-risk group for thromboembolic complications regardless of biologic therapy [2,14]. These findings support the importance of vigilant thromboprophylaxis in all patients with rheumatoid arthritis undergoing arthroplasty.

The question of optimal perioperative VTE prophylaxis in patients with rheumatoid arthritis undergoing arthroplasty remains unresolved. Current guidelines from the American Society of Hematology strongly recommend pharmacologic thromboprophylaxis for patients undergoing total hip or knee arthroplasty, with options including aspirin, low-molecular-weight heparin (LMWH), and direct oral anticoagulants (DOACs), and suggest DOACs over LMWH when anticoagulants are chosen [15]. These recommendations are derived from studies in the general arthroplasty population and are not tailored for patients with rheumatoid arthritis or those receiving JAK inhibitor therapy. Similarly, the 2022 ACR/AAHKS perioperative guideline defers to existing thromboprophylaxis protocols and does not address the potential VTE risk associated with JAK inhibitors [10]. This represents an important gap, as rheumatoid arthritis is independently associated with an approximately 1.5 to 2 fold increased baseline risk of VTE, which is likely further amplified in the perioperative setting [16,17]. The elevated absolute VTE rates observed in both cohorts of the study support the need for more tailored thromboprophylaxis strategies in this population. More broadly, VTE is a heterogeneous condition with outcomes that are shaped by patient- and treatment-related factors beyond the initial precipitant, as illustrated by large real-world registries in which concomitant pharmacologic therapies have been associated with differential prognosis among anticoagulated patients [18,19].

Whether patients with rheumatoid arthritis receiving JAK inhibitors should receive more intensive perioperative anticoagulation remains unresolved. The comparative effectiveness of aspirin versus DOACs for post-arthroplasty VTE prophylaxis remains debated, with meta-analytic data from randomized trials suggesting similar efficacy and large registry analyses demonstrating lower VTE rates with DOACs [20,21]. The intersection of RA-related inflammation, potential medication-associated thrombosis, and post-arthroplasty-related hypercoagulability suggests that an individualized approach to thromboprophylaxis is warranted. Because the present study did not compare specific prophylactic regimens, it cannot indicate whether any particular strategy is preferable in JAK inhibitor-treated patients. Prospective studies are needed to determine whether perioperative thromboprophylaxis should be individualized according to antirheumatic therapy and baseline thrombotic risk.

### Limitations

This study has several limitations. The number of observed VTE events was low, which limited statistical power and resulted in wide confidence intervals, making it difficult to exclude modest but clinically relevant differences between treatment groups. Medication exposure was defined using prescription records, and adherence or perioperative discontinuation could not be confirmed. This is particularly important given current practice of holding JAK inhibitors for several days prior to surgery, which likely reduced perioperative exposure and may have attenuated any true thrombotic signal. VTE outcomes were identified using diagnostic codes, introducing the possibility of misclassification. The single most important unmeasured confounder is perioperative thromboprophylaxis: the type, intensity, and duration of prophylaxis received by individual patients could not be determined, and differential prophylaxis between cohorts could account for part or all of the numerical difference in observed VTE rates. Our findings therefore cannot support any recommendation for more intensive anticoagulation in JAK inhibitor-treated patients. The use of a 365-day outcome window may also have captured events not directly attributable to the immediate postoperative period; however, shorter follow-up intervals produced suppressed event counts in the database, limiting our ability to evaluate early postoperative risk. Because exposure was defined during the 90 days preceding surgery whereas outcomes were ascertained over the subsequent year, the biological link between pharmacologic exposure and VTE necessarily weakens for events occurring remote from surgery, and the primary estimate should be interpreted as reflecting overall rather than strictly perioperative risk. Finally, residual confounding is inherent to retrospective database studies, as important factors such as RA disease activity, functional status, detailed obesity measures, and surgical complexity could not be accounted for. Subgroup analyses by individual JAK inhibitors and by procedure type were also not feasible because of limited sample size. Stratification by individual JAK inhibitor, by TNF inhibitor agent, and into separate DVT and PE outcomes was precluded because the corresponding event counts fell below the TriNetX cell-suppression threshold, and the pooling of agents with potentially distinct risk profiles is therefore a limitation. JAK inhibitors are also frequently used as second- or third-line therapy, so JAK inhibitor-treated patients may represent an older or more treatment-refractory population, and line of therapy could not be ascertained, representing potential confounding by indication. Hormone replacement therapy, an independent VTE risk factor, was likewise not captured among the matching covariates. Our findings should therefore be interpreted as exploratory and hypothesis-generating.

## 5. Conclusions

Among patients with rheumatoid arthritis undergoing total knee or total hip arthroplasty, preoperative use of JAK inhibitors was not associated with a statistically significant difference in postoperative venous thromboembolism risk compared with TNF inhibitors. Although event rates were numerically higher in the JAK inhibitor cohort, these differences did not reach statistical significance and should be interpreted cautiously given the small number of events and wide confidence intervals. Rather than demonstrating equivalence, these findings provide hypothesis-generating data and underscore the need for larger studies to better define the magnitude and timing of postoperative thromboembolic risk associated with JAK inhibitor therapy. Clinically, these findings do not justify changes to current ACR/AAHKS perioperative management or the adoption of more intensive anticoagulation in JAK inhibitor-treated patients; rather, they support continued guideline-concordant thromboprophylaxis for all RA patients undergoing arthroplasty and identify an adequately powered prospective study as the priority for resolving the residual uncertainty.

## Figures and Tables

**Table 1:** Baseline Characteristics Before and After Propensity Score Matching. Values are presented as mean ± standard deviation or number (percentage). JAK = Janus kinase; TNF = tumor necrosis factor; SD = standard deviation; SMD = standardized mean difference. An SMD <0.10 was considered evidence of adequate balance between cohorts. Counts for low-frequency variables may be masked or rounded in accordance with TriNetX privacy policies.

Characteristic	TNF Before (*n* = 1299)	JAK Before (*n* = 305)	SMD	TNF After (*n* = 304)	JAK After (*n* = 304)	SMD
**Demographics**						
Age, years (mean ± SD)	61.3 ± 11.5	60.0 ± 11.5	0.11	60.1 ± 12.1	60.0 ± 11.4	0.00
Women, n (%)	969 (74.6)	251 (82.3)	0.19	247 (81.3)	250 (82.2)	0.03
Men, n (%)	329 (25.3)	54 (17.7)	0.19	57 (18.8)	54 (17.8)	0.03
White race, n (%)	963 (74.1)	202 (66.2)	0.17	199 (65.5)	202 (66.4)	0.02
Asian, n (%)	25 (1.9)	13 (4.3)	0.14	13 (4.3)	13 (4.3)	0.00
American Indian or Alaska Native, n (%)	12 (0.9)	10 (3.3)	0.16	10 (3.3)	10 (3.3)	0.00
**Comorbidities**						
Hypertension, n (%)	560 (43.1)	108 (35.4)	0.16	99 (32.6)	108 (35.5)	0.06
Type 2 diabetes mellitus, n (%)	175 (13.5)	37 (12.1)	0.04	33 (10.9)	37 (12.2)	0.04
Disorders of lipoprotein metabolism (hyperlipidemia), n (%)	349 (26.9)	75 (24.6)	0.05	70 (23.0)	75 (24.7)	0.04
Overweight and obesity, n (%)	202 (15.6)	50 (16.4)	0.02	46 (15.1)	50 (16.4)	0.04
Personal history of nicotine dependence, n (%)	86 (6.6)	27 (8.9)	0.08	24 (7.9)	26 (8.6)	0.02
Nicotine dependence, n (%)	67 (5.2)	12 (3.9)	0.06	13 (4.3)	12 (3.9)	0.02
**Prior Cardiovascular and Thrombotic History**						
Other venous embolism and thrombosis, n (%)	10 (0.8)	10 (3.3)	0.18	10 (3.3)	10 (3.3)	0.00
Pulmonary embolism, n (%)	10 (0.8)	10 (3.3)	0.18	10 (3.3)	10 (3.3)	0.00
Acute myocardial infarction, n (%)	10 (0.8)	10 (3.3)	0.18	10 (3.3)	10 (3.3)	0.00
Cerebral infarction, n (%)	10 (0.8)	10 (3.3)	0.18	10 (3.3)	10 (3.3)	0.00
**Medications**						
Glucocorticoids, n (%)	486 (37.4)	122 (40.0)	0.05	110 (36.2)	121 (39.8)	0.07
Methotrexate, n (%)	334 (25.7)	52 (17.0)	0.21	53 (17.4)	52 (17.1)	0.01
Anticoagulants, n (%)	134 (10.3)	26 (8.5)	0.06	22 (7.2)	26 (8.6)	0.05
Antiplatelet agents, n (%)	133 (10.2)	28 (9.2)	0.04	23 (7.6)	28 (9.2)	0.06

**Table 2:** Primary Outcome: Venous Thromboembolism. JAK = Janus kinase; TNF = tumor necrosis factor; CI = confidence interval. Risk ratio and odds ratio are presented for JAK inhibitor use relative to TNF inhibitor use.

Outcome	JAK Inhibitor Cohort, n/N (%)	TNF Inhibitor Cohort, n/N (%)	Absolute Risk Difference, % (95% CI)	Risk Ratio (95% CI)	Odds Ratio (95% CI)	*p*-Value
Venous thromboembolism	17/284 (5.99)	11/288 (3.82)	2.17 (−1.37 to 5.70)	1.57 (0.75 to 3.29)	1.60 (0.74 to 3.49)	0.230

## Data Availability

The data was obtained under from the TriNetX Global Collaborative Network (TriNetX, LLC) and are not publicly available. Access is available to researchers through a subscription agreement with TriNetX. Aggregate results are available from the corresponding author upon reasonable request.
